# Prognostic Immunophenotyping Clusters of Clear Cell Renal Cell Carcinoma Defined by the Unique Tumor Immune Microenvironment

**DOI:** 10.3389/fcell.2021.785410

**Published:** 2021-12-06

**Authors:** Wenhao Xu, Aihetaimujiang Anwaier, Chunguang Ma, Wangrui Liu, Xi Tian, Jiaqi Su, Wenkai Zhu, Guohai Shi, Shiyin Wei, Hong Xu, Yuanyuan Qu, Dingwei Ye, Hailiang Zhang

**Affiliations:** ^1^ Department of Urology, Fudan University Shanghai Cancer Center, Shanghai Medical College, Fudan University, Shanghai, China; ^2^ Affiliated Hospital of Youjiang Medical University for Nationalities, Baise, China

**Keywords:** clear cell renal cell carcinoma, immunophenotyping, immune checkpoint therapies, prognosis (carcinoma), tumor microenvironment, machine-learning algorithms

## Abstract

**Background:** The tumor microenvironment affects the occurrence and development of cancers, including clear cell renal cell carcinoma (ccRCC). However, how the immune contexture interacts with the cancer phenotype remains unclear.

**Methods:** We identified and evaluated immunophenotyping clusters in ccRCC using machine-learning algorithms. Analyses for functional enrichment, DNA variation, immune cell distribution, association with independent clinicopathological features, and predictive responses for immune checkpoint therapies were performed and validated.

**Results:** Three immunophenotyping clusters with gradual levels of immune infiltration were identified. The intermediate and high immune infiltration clusters (Clusters B and C) were associated with a worse prognosis for ccRCC patients. Tumors in the immune-hot Clusters B and C showed pro-tumorigenic immune infiltration, and these patients showed significantly worse survival compared with patients in the immune-cold Cluster A in the training and testing cohorts (*n* = 422). In addition to distinct immune cell infiltrations of immunophenotyping, we detected significant differences in DNA variation among clusters, suggesting a high degree of genetic heterogeneity. Furthermore, expressions of multiple immune checkpoint molecules were significantly increased. Clusters B and C predicted favorable outcomes in 64 ccRCC patients receiving immune checkpoint therapies from the FUSCC cohort. In 360 ccRCC patients from the FUSCC validation cohort, Clusters B and C significantly predicted worse prognosis compared with Cluster A. After immunophenotyping of ccRCC was confirmed, significantly increased tertiary lymphatic structures, aggressive phenotype, elevated glycolysis and PD-L1 expression, higher abundance of CD8^+^ T cells, and TCRn cell infiltration were found in the immune-hot Clusters B and C.

**Conclusion:** This study described immunophenotyping clusters that improved the prognostic accuracy of the immune contexture in the ccRCC microenvironment. Our discovery of the novel independent prognostic indicators in ccRCC highlights the relationship between tumor phenotype and immune microenvironment.

## Highlights

• This study identified immunophenotyping clusters that show the prognostic accuracy of the immune contexture in the ccRCC microenvironment.
• The immune-hot Clusters B and C showed a transcriptional signature indicative of pro-tumorigenic immune infiltration and show significantly worse survival compared with the immune-cold Cluster A.
• Our discovery of the novel independent prognostic indicators in ccRCC highlights the relationship between tumor phenotype and the immune contexture.

## Introduction

Renal cell carcinoma (RCC) is one of the most common malignant tumors of the genitourinary system, accounting for about 5% of all new cases in adult male patients and 3% of new cases in female patients (Siegel et al., 2020). RCC is classified into three main histological subtypes, including clear cell RCC (ccRCC), papillary RCC, and chromophobe RCC, which are considered to arise from different cell types in the nephron (Linehan, 2012; Moch et al., 2016; Hsieh et al., 2017). ccRCC is the predominant subtype of RCC, accounting for 70%–85% of all RCC patients, and is highly malignant (Gerlinger et al., 2012; Miller et al., 2019). Although classic histological heterogeneity has been widely accepted in the research and treatment of RCC, the latest advances in genomic technologies have revealed different clinically relevant molecular subtypes, which have aided in elucidating the molecular basis of RCC as well as mechanisms underlying the occurrence and development of RCC. These findings have led to improved targeted treatment measures for patients with RCC.

Although satisfactory prognosis of localized ccRCC can be achieved through radical or nephron-preserving nephrectomy, metastatic or advanced ccRCC requires systematic treatment strategies (Hofmann et al., 2020). The standard first-line systematic treatment for metastatic or advanced ccRCC includes tyrosine kinase inhibitors (TKIs) such as sunitinib or pazopanib that target the vascular endothelial growth factor receptor (VEGFR) (Motzer et al., 2007; Escudier et al., 2019; Porta et al., 2019). While anti-angiogenic drugs effectively inhibit tumor proliferation and prolong the overall survival of low-risk ccRCC patients, the objective response rate of these treatments is unsatisfactory (Heng et al., 2009; Motzer et al., 2009; Sternberg et al., 2010). Recently, new immune checkpoint therapies (ICTs) represented by PD-1/PD-L1 and CTLA-4 inhibitors have been demonstrated in clinical trials as treatments for ccRCC (Motzer et al., 2019). However, because of the high tumor heterogeneity and low somatic mutation burden of ccRCC, an accurate and effective model for the prediction of clinical treatment is required (Grimm et al., 2019; Kotecha et al., 2019).

To reveal the underlying molecular heterogeneity of ccRCC, Brannon et al. analyzed the transcriptional expression profiles of 48 ccRCC patients and identified two molecular subtypes of ccRCC (ccA and ccB) (Brannon et al., 2010). A meta-analysis of large-scale ccRCC subsequently confirmed this classification and identified a new subtype (cluster_3) associated with von Hippel-Lindau (VHL) gene mutation (Brannon et al., 2012). The Cancer Genomic Atlas (TCGA) analyzed extensive transcriptional profiles of ccRCC patients and recognized four molecular subtypes of ccRCC (m1–m4) with various somatic mutations and distinct prognosis (Cancer Genome Atlas Research Network, 2013). In the metastatic setting, four molecular subtypes (ccrcc1–4) related to the objective response rate and overall survival (OS) of angiogenesis inhibitors sunitinib and pazopanib were identified. These subtypes differ not only in mRNA expression profiles but also in somatic mutations, methylation status, and immune cell infiltration in the tumor microenvironment (TME) (Beuselinck et al., 2015).

The cells and molecules in the TME are in a process of dynamic change, reflecting the evolutionary nature of cancer, and together these factors promote the proliferation, apoptosis, metastasis, and immune escape of cancer cells (Fridman et al., 2017). A large amount of evidence has shown that not only does the efficacy of immunotherapy depend on activation of the tumor immune microenvironment, but the efficacy of traditional targeted therapy also depends on the strength of the individual antitumor immune response (Fridman et al., 2017; Kawakami et al., 2017). ICTs combined with TKIs effectively inhibit the occurrence and development of advanced ccRCC and induces the normalization of antitumor immunity, which depends on a deep understanding of the interaction between tumor cells and TME (Chen and Mellman, 2017). ccRCC patients are mainly of immune infiltrating type (73%), enriched with antitumor M1 macrophages, activated CD^4+^ memory T cells, and activated NK cells, but the immune contexture failed to accurately predict the efficacy of anti-PD-1 therapy and mTOR inhibitors (Braun et al., 2020). Our previous studies identified a relationship between immune component infiltration in TME and prognosis of ccRCC patients as well as TME regulatory cytokines and emphasized the role of TME-related markers in the prognosis of ccRCC patients; our findings also supported the use of precise immunotherapy for high-risk ccRCC patients (Xu et al., 2019). Therefore, exploring the underlying mechanism of TME-driven tumorigenesis and development is of great significance for developing potential therapeutic prediction models, improving the effectiveness of existing treatment strategies, and discovering novel precise targets for ccRCC treatment.

The TME affects the development, occurrence, and treatment resistance of cancers including ccRCC. However, how the immune cell mixture interacts with the cancer phenotype and affects pathogenesis remains unclear. We therefore sought to identify novel immunophenotyping subtypes of ccRCC that may help improve the prognostic accuracy and of immune contexture in the ccRCC microenvironment based on large-scale data, with the aim of providing a theoretical basis for the development of individual precise treatment strategies of ICTs.

## Methods

### Data download and preprocessing from the training, testing, and validation cohorts

The RNA sequencing data of 531 ccRCC patients were obtained from The Cancer Genome Atlas (TCGA, https://portal.gdc.cancer.gov) with gene IDs converted from Ensembl ID to gene symbol matrix. The FPKM gene expression profile was measured experimentally using the Illumina HiSeq 2000 RNA Sequencing platform by the University of North Carolina TCGA genome characterization center. Level 3 data were downloaded from the TCGA data coordination center, with available clinicopathological and survival data. In addition, the phenotypic and clinical data of 531 ccRCC patients were obtained from the TCGA training cohort and 91 ccRCC patients from the International Cancer Genome Consortium (ICGC, https://dcc.icgc.org/) testing cohort. A total of 770 genes were downloaded from The nCounter^®^ PanCancer Immune Profiling panel (https://www.nanostring.com/products/ncounter-assays-panels/oncology/pancancer-immune-profiling/) and 758 immune genes were matched in the TCGA database for further analysis (Cesano, 2015).

A total of 64 ccRCC patients receiving ICTs alone or combined with TKI treatments were enrolled from the Fudan University Shanghai Cancer Center (FUSCC, Shanghai, China) cohort. A total of 360 ccRCC patients with long-term follow-up information from FUSCC cohort were enrolled as prognostic validation cohort of immunophenotyping clusters.

### Construction of immune-phenotyping and subgroup analysis

To identify prognostic clusters based on tumor immune microenvironment features, we enrolled large-scale ccRCC cohorts with available RNA-seq data and constructed immune clusters based on 758 immune genes; the association between the immune clusters and tumor heterogeneity was then explored.

The correlation matrix was calculated based on the expression of 758 genes. The R “pheatmap” package was utilized to hierarchically cluster the correlation matrix of patients, where the correlation was used as the clustering distance and ward.D as a link (Galili et al., 2018). Besides, cutree function was utilized to identify subgroups of ccRCC samples. In order to determine the optimal number of clusters for each queue, “Cluster” package of R software was used to perform a contour analysis on KMeans. The subgroup myeloid infiltration score, dryness index score, immune score, and mutation were calculated, and the survival analysis of the subgroups was performed using the Kaplan–Meier method.

### Construction of a classifier to predict immunophenotyping clusters

Next, the immunophenotyping clusters were established using a machine-learning algorithm and used to categorize ccRCC patients for easier clinical application. Immune subgroups were predicted by binomial logistic regression using R software “glmnet” package (Engebretsen and Bohlin, 2019), which could assign samples divided into groups without unsupervised clustering. The risks score of 28 hub immune genes used for predicting immunophenotying clusters of ccRCC are shown in the supplementary tables and mentioned in the results section.

The binomial distribution was used to develop a logistic regression predicting the classification based on the gene expression profile. Besides, operating characteristic curve (ROC) curves were plotted using the R software “pROC” package. The area under the curve (AUC) is used to evaluate the specificity and sensitivity of the clusters. Logistic regression coefficients were used to calculate the risk scores of each ccRCC sample.

### Assessment of immune cell infiltration

To assess the absolute proportion of 22 infiltrating immune cells in ccRCC samples from TCGA, we performed the CIBERSORT algorithm (Chen et al., 2018). As a deconvolution algorithm, CIBERSORT utilizes a set of reference gene expression values (547 genes) to predict the proportion of immune cell types using support vector regression. In order to evaluate the reliability of the deconvolution method, the “CIBERSORT” R package provides a *p* value for each sample using a default feature matrix with perm = 100 times for analysis.

### Single sample gene set enrichment analysis

The GSVA Bioconductor R package was used for genome functional enrichment analysis. The C2 and hallmark datasets were downloaded from the MSigDB database (https://www.gsea-msigdb.org/gsea/msigdb), collecting a variety of function annotations including epithelial–mesenchymal transitions (EMTs), stem cell proliferation, and cell cycle-related pathways.

### Assessment of DNA variation

The single-nucleotide polypeptide (SNP) data and MAF profile of ccRCC patients were downloaded from FireBrowse (http://firebrowse.org/) and analyzed using the R “maftools” package. The copy number variation (CNV) data with level 3 was downloaded from Broad Institute and analyzed using the GISTIC2 module in the GenePattern cloud platform, with Reference Genome Fileselects selecting “hg19.”

### Assessment of immunotherapy efficacy and long-term prognostic implications

Differential immune checkpoint molecular expression, including PD-L1, PD-L2, LAG-3, IL-8, PDCD1, CTLA4, and TIGIT, and PBRM1 expression were enrolled between immunophenotyping clusters. Then, we enrolled 35 ccRCC patients receiving ICTs from the CA209-009 cohort with gene-specific enrichment in clinical benefits. Moreover, RT-qPCR was utilized to evaluate the relative expression of hub genes and immunophenotyping clusters. A total of 64 ccRCC patients receiving ICTs alone or combined with TKI treatments were enrolled from the Fudan University Shanghai Cancer Center (FUSCC, Shanghai, China) cohort. A total of 360 ccRCC patients with long-term follow-up information from the FUSCC cohort were enrolled as a prognostic validation cohort of immunophenotyping clusters.

### Evaluation of tumor immune microenvironment characterizations

The tertiary lymphoid structure (TLS) was assessed using hematoxylin–eosin (HE) staining, and immunohistochemistry (IHC) was utilized to evaluate the expression level of Ki-67 (ab15580; Abcam), Glut-1 (ab115730; Abcam), and PD-L1 (ab205921; Abcam) according to procedures, as previously described (Wang et al., 2020). Then, an opal multispectral was used to identify differential immune cell infiltration. CD3 (Kit-0003, Maxim, Shenzhen, China), CD4, (RMA-0620, Maxim, China), CD8 (RMA-0514, Maxim, China), CK (Kit-0009, Maxim, China), FoxP3 (98377, CST), and PD-L1 (13684, CST) antibodies were added to the slide and incubated overnight in a humidified chamber at 4°C. An HRP-labeled goat anti-rabbit/mouse secondary antibody was added dropwise and incubated at 37°C for 30 min. Finally, the slices are imaged and quantitatively analyzed on a multispectral imaging system (Vectra^®^ Polaris™, Shanghai, China).

### Statistical analysis

In the statistical analyses, the Wilcox test was used to compare the differences between the two groups of samples. The survminer of the R package and X-tile, a single-function software developed by Yale University, were utilized to take the best cutoff value for all survival analyses. The survival curve was analyzed by Kaplan–Meier, and the log-rank test was used to determine the significance of the difference. The receiver operating characteristic (ROC) is used to evaluate prediction sensitivity and specificity of immunophenotyping clusters in the disease progression, and the AUC is used to evaluate the specificity and sensitivity of the model.

## Results

**FIGURE 1:**
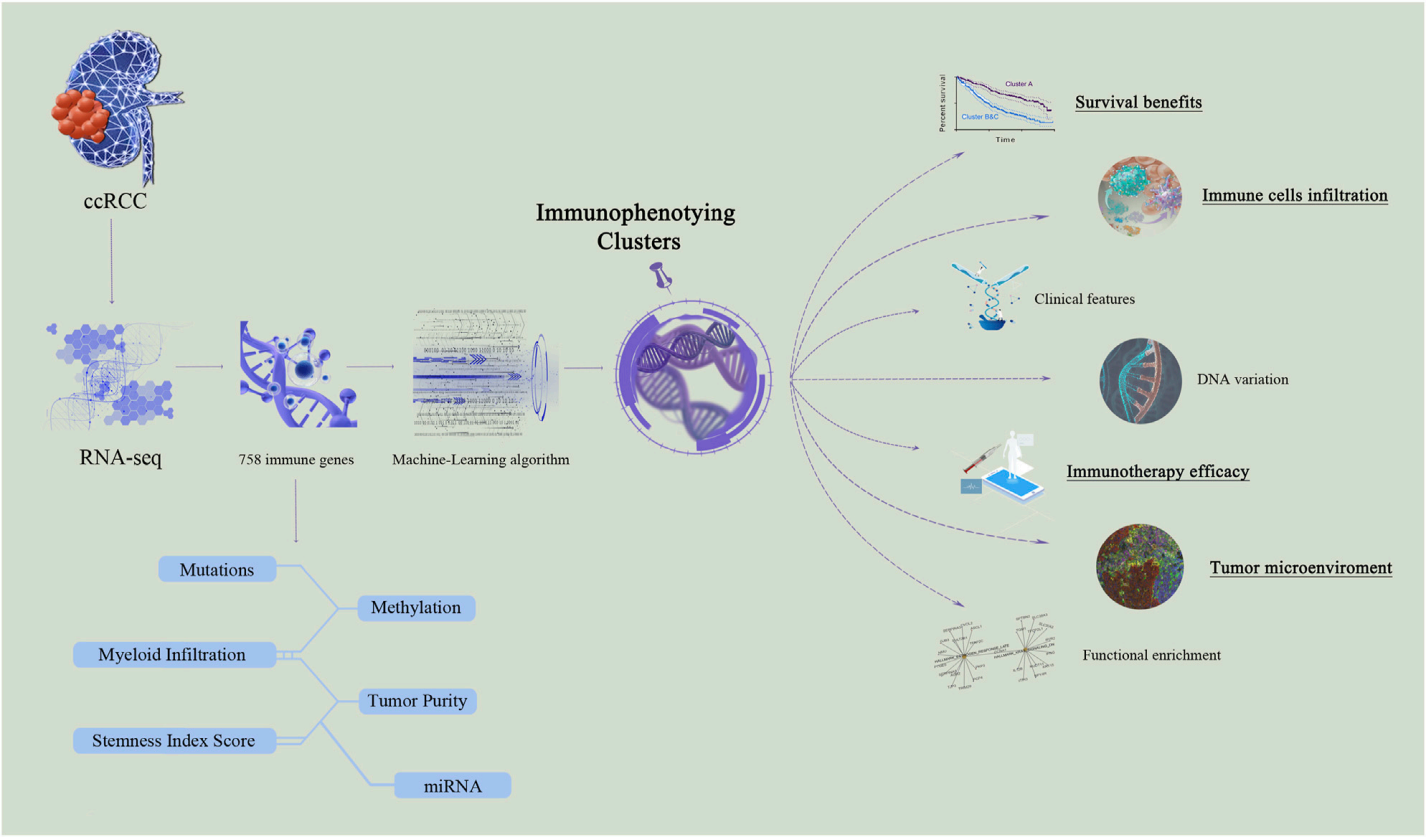
Computational and experimental workflow for immunophenotyping clusters.

The TME has been implicated in various malignant biological processes, including carcinogenesis, irregular cellular metabolism, and abnormal immune regulation. This study was conducted in three phases to explore immunophenotyping clusters of ccRCC, and the study flow is shown in Figure 1. First, we enrolled large-scale ccRCC cohorts with available RNA-seq data and constructed immune clusters based on 758 immune genes; the association between the immune clusters and tumor heterogeneity was then explored. Second, the immunophenotyping clusters were established using a machine-learning algorithm and used to categorize ccRCC patients; the clusters showed differences in DNA variation, functional enrichment, clinical features, survival benefits, immunotherapy responses, immune cell distribution, and the tumor immune microenvironment *in silico*. Third, immunophenotyping clusters were used to estimate TME characterizations, long-term prognosis, and predictive responses to ICTs for ccRCC patients from public to real-world validation cohorts.

### Screening and initial construction of subgroups based on 758 immune genes

**FIGURE 2:**
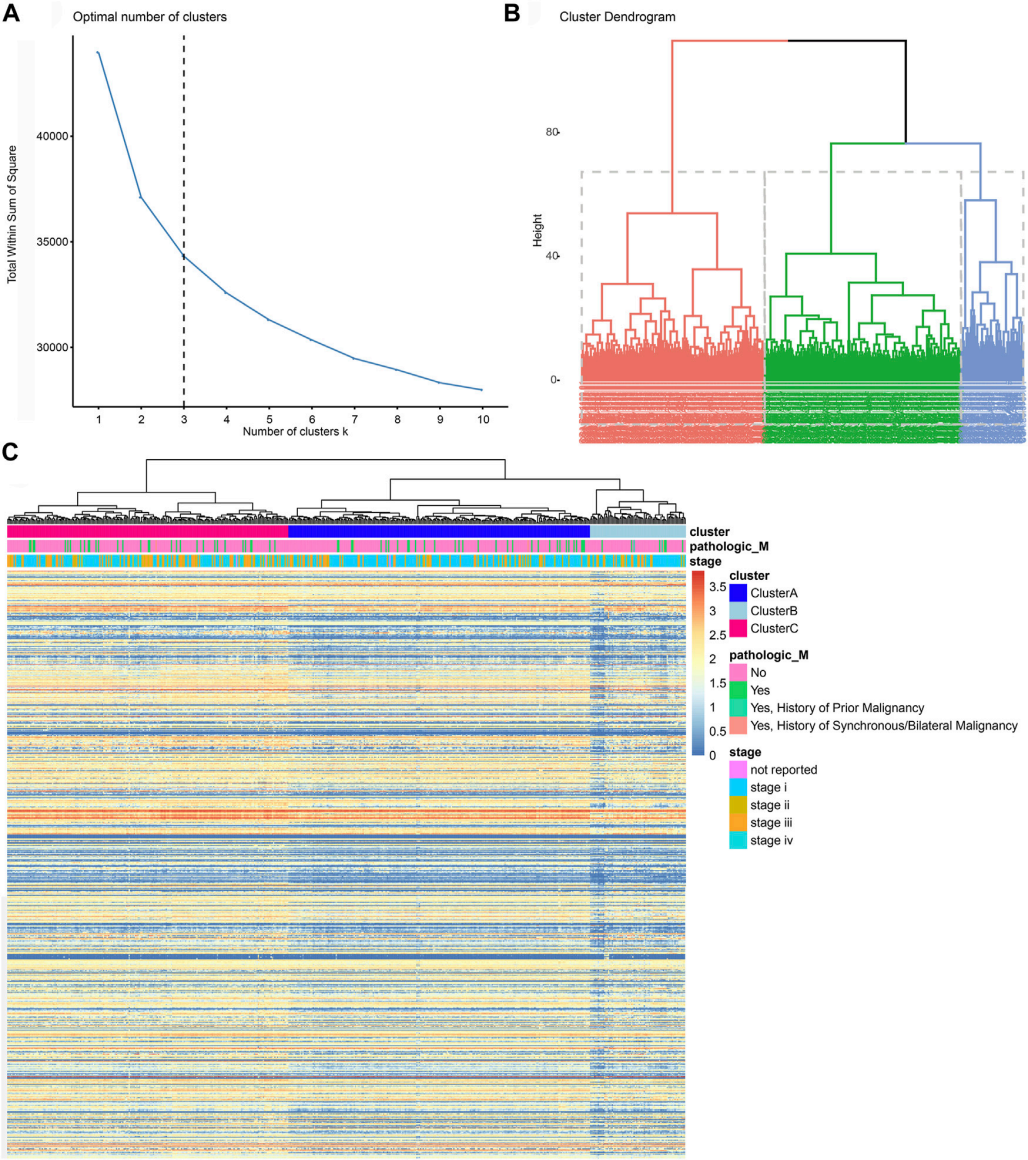
Screening and initial construction of subgroups based on 758 immune genes. **(A, B)** Hierarchically clustering of 758 immune genes from the nCounter^®^ PanCancer Immune Profiling panel. **(C)** Construction of subgroups based on 758 immune genes enrolled traditional clinicopathological indicators of 531 ccRCC samples from the TCGA database.

First, we matched immune genes in the nCounter^®^ PanCancer Immune Profiling panel with those in transcriptome data from the TCGA database and obtained the expression profile of 758 immune genes (Supplementary Table S1A,B). We then obtained the correlation matrix, hierarchically clustered the correlation matrix of ccRCC patients, and confirmed three subgroups (Clusters A, B, and C) as the optimal clustering (Figures 2A,B; Supplementary Table S1C). We enrolled traditional clinicopathological indicators of 531 ccRCC samples from the TCGA database and found that the expression of immune genes in Cluster C patients was markedly higher than that of the other two subgroups, and the expression of immune genes in Cluster A was at an intermediate level (Figure 2C).

### Relation between immune clusters and tumor heterogeneity of ccRCC

**FIGURE 3:**
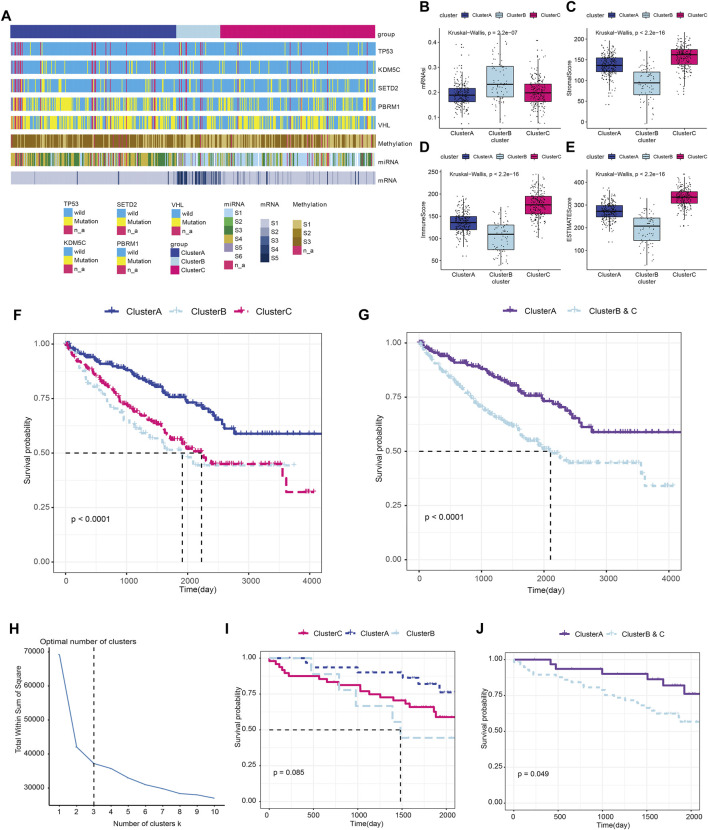
Immune clusters predict tumor heterogeneity and outcomes of ccRCC in training and testing cohorts. **(A)** Association between the immune clusters and tumor heterogeneity in genetic and epigenetic levels. **(B–E)** The myeloid infiltration score (StromalScore), immune score (ImmuneScore), tumor purity (ESTIMATEScore), and stemness index score (mRNAsi) based on RNA expression data from the TCGA database were measured among subgroups using the Kruskal–Wallis test. **(F)** Prognosis was compared in the three immune subgroups in the TCGA-ccRCC cohort. **(G)** Prognosis was compared between Cluster A and the newly defined Clusters B and C in the training cohort. **(H–J)** Prognostic value of the immune clusters in 91 ccRCC patients from the ICGC testing cohort.

We next analyzed the immune clusters and tumor heterogeneity information at genetic and epigenetic levels (Figure 3A). The results indicated that VHL and PBRM1 genes were the most frequently mutated genes in ccRCC, and Cluster A showed a relatively higher mutation rate than Clusters B and C. We also performed subgroup analysis of ccRCC and found significantly differential heterogeneity in methylation, miRNA, and mRNA levels among the three subgroups (*p* < 0.05). Next, we measured the myeloid infiltration score (StromalScore), immune score (ImmuneScore), tumor purity (ESTIMATEScore), and stemness index score (mRNAsi) among subgroups based on RNA expression data from the TCGA database (Figures 3B–E; Supplementary Table S2A). Overall, these results revealed significant differences in tumor heterogeneity among the three immune clusters using the Kruskal–Wallis test (*p* < 0.001).

### Immune clusters predict outcomes of ccRCC in training and testing cohorts

We analyzed the prognosis of the three immune subgroups and found that patients in Clusters B and C showed similar outcomes and significantly poorer survival compared with Cluster A (*p* < 0.0001; Figure 3F). We then combined Clusters B and Clusters C into the newly defined Clusters B and C (Supplementary Table S2B) and compared the prognosis between Cluster A and Cluster B and C. The results revealed markedly poor survival in Clusters B and C compared with Cluster A (*p* < 0.0001; Figure 3G). To validate the prognostic value of the immune clusters, we enrolled 91 ccRCC patients with available RNA-seq data from the ICGC cohort (Figure 3H; Supplementary Table S3). The clinical outcomes among three subgroups showed no significant differences (*p* = 0.085; Figure 3I). However, significantly poorer prognosis was observed in Clusters B and C compared with Cluster A (*p* = 0.049; Figure 3J).

### Construction of a classifier to predict immune subgroups using machine-learning algorithms

**FIGURE 4:**
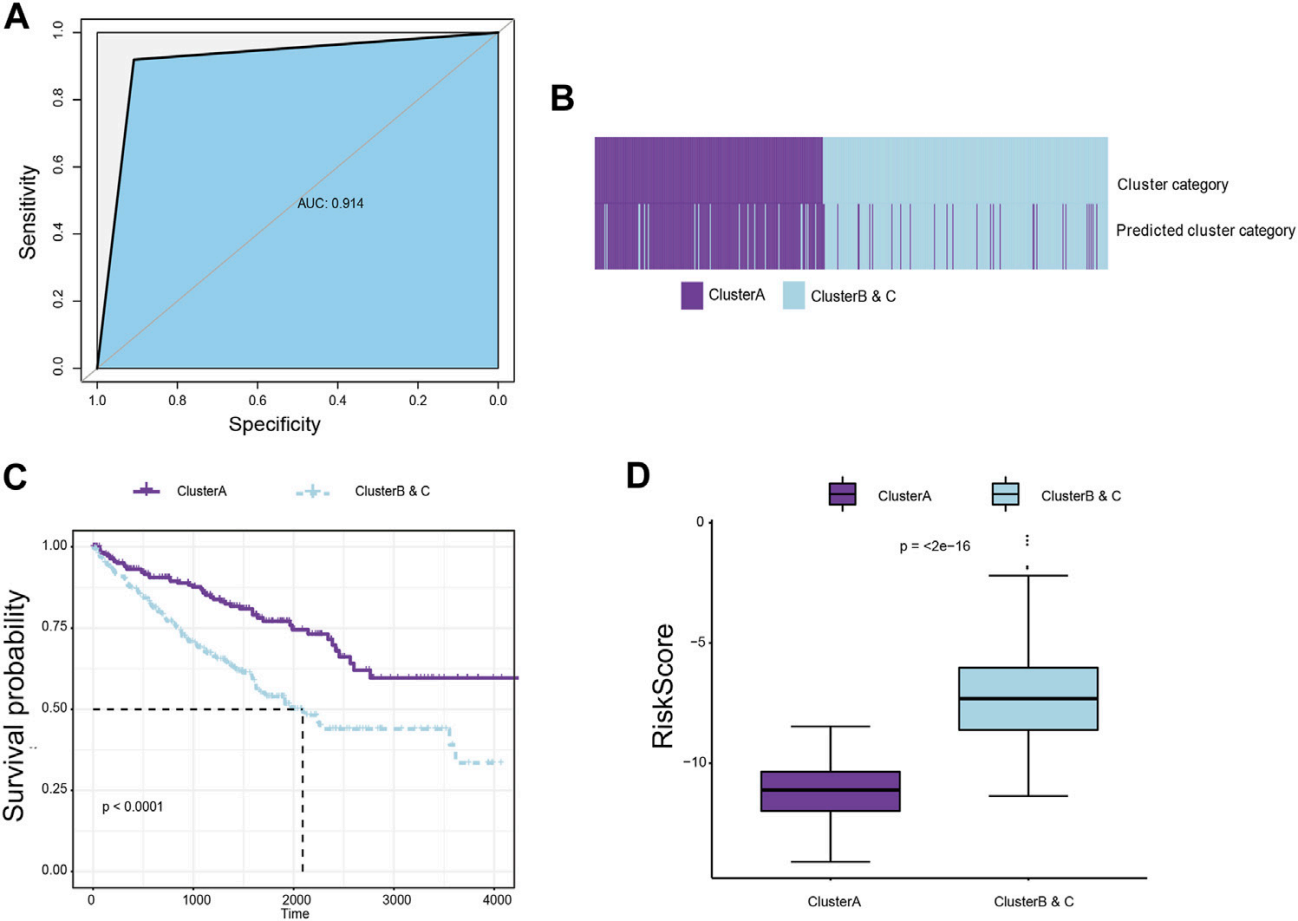
Construction of a classifier to predict immune subgroups using machine-learning algorithms. **(A)** Machine-learning algorithms were used to develop a simple predictor to predict immune clusters, thereby randomly assigning all samples to the group with poor or good prognosis until the best prediction efficiency is obtained. A total of 26 hub immune genes were identified for the prognostic predictor for subgroup classification. **(B)** For the training set samples, immunophenotyping clustering was used to determine whether the samples belonged to Cluster A or Clusters B and C. **(C)**. Survival difference between immunophenotyping clusters of ccRCC patients in the training cohort. **(D)** The logistic regression coefficient was further used to calculate the risk score of each sample.

To further explore differences between the subgroups and improve the clinical translational efficacy, we implemented a series of machine-learning algorithms to develop a simple predictor predicting immune clusters, thereby randomly assigning all samples to the group with poor or good prognosis until the best prediction efficiency is obtained. A total of 28 hub immune genes were identified for the prognostic predictor for subgroup classification, named immunophenotyping clusters (AUC = 0.914; Figure 4A; Supplementary Table S4A). As shown in Supplementary Figure S3, we summarized prognostic implications of significant hub immune genes in ccRCC. The K–M survival analysis emphasized the prognostic significance of SOCS1, SAA1, TLR3, PRKCE, HNRNPA2B1, PDCD1, IL1R2, FCGR1A, CD36, CASP3, CARD11, and BCL2 as cancer-promoting factors of ccRCC. For the training set samples, immunophenotyping clustering was used to analyze whether the samples belonged to Cluster A or Clusters B and C. Through model prediction, 91.9% of the samples were assigned to Cluster A, and 90.8% of samples were assigned to clusters B and C (Figure 4B; Supplementary Table S4B). We observed significant differences in survival between the immunophenotyping clusters of ccRCC patients (*p* < 0.0001; Figure 4C). The logistic regression coefficient was further used to calculate the risk score of each sample; the risk score of Clusters B and C was significantly higher than that of Cluster A (*p* < 2e-16; Figure 4D; Supplementary Table S4C).

### Clinicopathological characteristics of immunophenotyping clusters

Next, we analyzed the differences of various clinical indicators in the different immunophenotyping clusters. Interestingly, we found a significantly decreased tumor purity in Clusters B and C, which showed with worse prognosis, compared with Cluster A (*p* = 2.9e-08; Supplementary Figure S2A). The methylation and mRNA expression levels of CDKN2A in Clusters B and C were significantly higher than in Cluster A (*p* < 1e-04; Supplementary Figure S2B,C), while the total number of mutations between the two clusters did not show significant differences (*p* = 0.16; Supplementary Figure S2D).

We also analyzed other phenotypic indicators between the immunophenotyping clusters, such as sex, age, tumor stage, smoking status, microsatellite instability (MSI), resection, or biopsy site. We found significantly more ccRCC patients from Clusters B and C in the smoker group and fewer patients from Clusters B and C in the non-smoker group (*p* < 0.05; Supplementary Figure S2E). Clusters B and C also showed a significantly elevated risk score compared with Cluster A regardless of smoking status (*p* < 0.001; Supplementary Figure S2F). Significantly higher numbers of male patients were present in Clusters B and C (*p* < 0.05; Supplementary Figure S2G). MSI and age did not show differences in the two subgroups (*p* < 0.05; Supplementary Figure S2H,I). As shown in Supplementary Figure S2J, more patients from Clusters B and C were in advanced stages compared with patients in Cluster A.

### Immune cell infiltration analysis of immunophenotyping clusters

**FIGURE 5:**
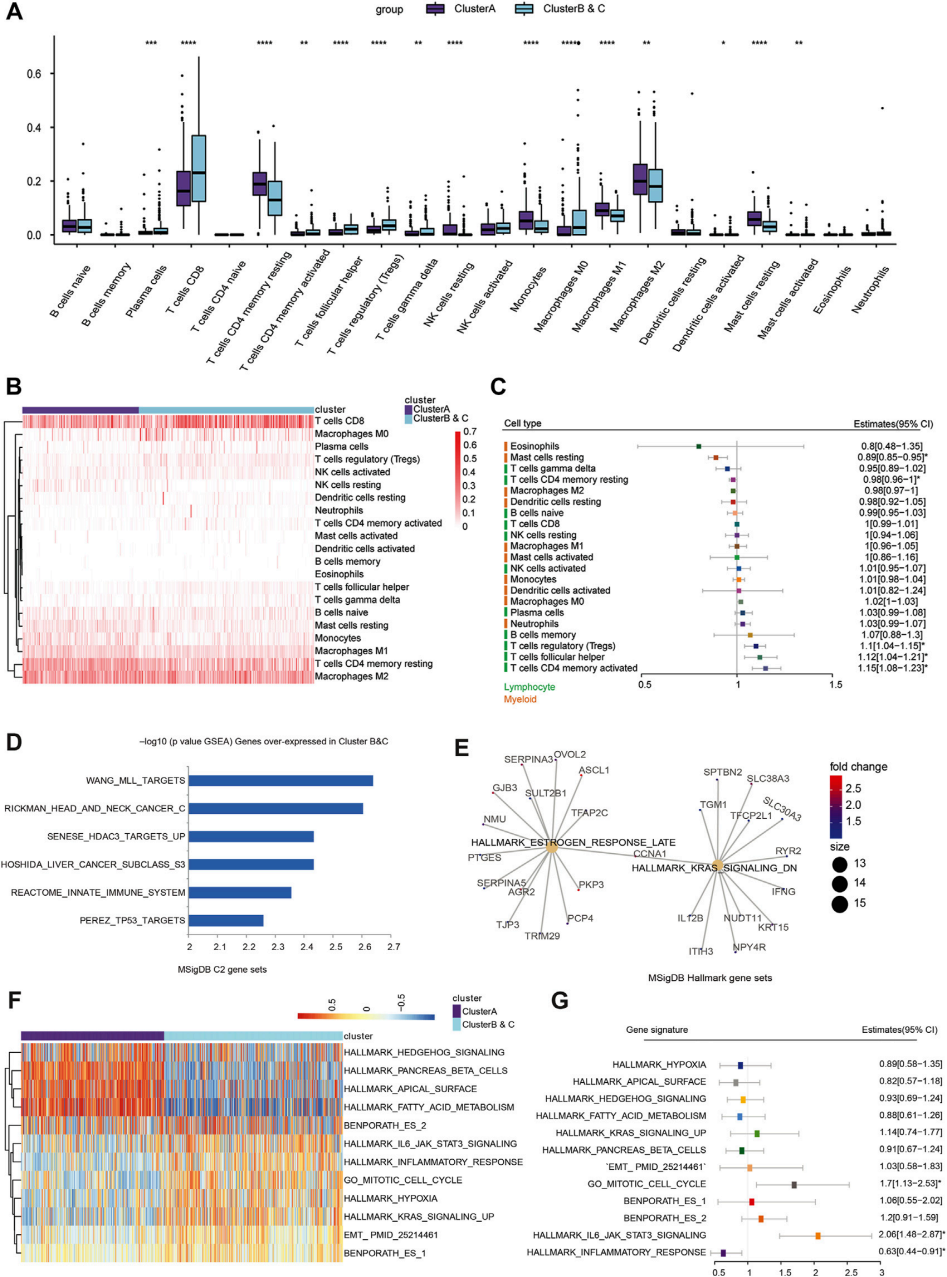
Immune cell infiltration and function enrichment analysis of immunophenotyping clusters. **(A, B)** The CIBERSORT algorithm was performed to analyze the absolute proportion of 22 infiltrating immune cells and explore differential immune cell distribution in ccRCC samples from TCGA. **(C)** Prognostic value of lymphocyte-derived and myeloid-derived immune cell infiltration using univariate Cox regression analysis in a forest plot. **(D, E)** To evaluate the differences in biological function between the two immunophenotyping clusters, the Wilcox test was used to identify DEGs in the two immunophenotyping clusters. According to the screening criteria of |log_2_FC|>1 and adj.Pvalue<0.01 (Supplementary Materials), GSEA was used to explore the functional annotations of upregulated DEGs in Clusters B and C. **(F)** The GSVA algorithm suggested significantly enriched hallmarks in Cluster A and Clusters B and C. **(G)** Generalized linear model Cox regression model was used to test the contribution of each function to Clusters B and C and Cluster A.

To explore differences in immune cell distribution, the CIBERSORT algorithm was used to analyze the absolute proportion of 22 infiltrating immune cells in ccRCC samples from the TCGA database (Supplementary Table S5). There were significant differences in immune cell infiltration between the immunophenotyping clusters, especially in plasma cells, CD4 memory resting T cells, follicular helper T cells, regulatory T cells (Tregs), resting dendritic cells, and resting mast cells (Figures 5A,B). A significant increase of CD8^+^ T cells was found in Clusters B and C compared with Cluster A. We also assessed the distribution of risk ratios in each immune cell infiltration and identified the prognostic implications. As shown in Supplementary Figure S3, there were significantly differences in survival associated with CD4 memory-activated T cell, follicular helper T cell, Tregs, CD4 memory resting T cell, and resting mast cell infiltration. In addition, we examined the prognostic value of lymphocyte-derived and myeloid-derived immune cell infiltration using univariate Cox analysis, as shown in Figure 5C. The results suggested that elevated lymphocyte-derived CD4^+^ memory activated T cells (HR = 1.15), Tfh cells (HR = 1.12), and Treg cells (HR = 1.10) were significantly correlated with poor outcomes for ccRCC patients, while elevated myeloid-derived resting mast cells (HR = 0.89) predicted favorable prognosis for ccRCC patients.

### Function enrichment analysis of immunophenotyping clusters

To evaluate the differences in biological function between the two immunophenotyping clusters, we performed genomic function enrichment analysis using GSVA. The Wilcox test was used to identify differentially expressed genes (DEGs) in the two immunophenotyping clusters. According to the screening criteria of |log_2_FC|>1 and adj.pvalue<0.01, we screened out 1,045 DEGs, with 157 genes upregulated in Cluster A and 888 genes upregulated in Cluster B and C (Supplementary Table S6A). GSEA was used to explore the functional annotations of the upregulated DEGs in Clusters B and C, and these DEGs were significantly enriched in C2 functions such as TP53 targets, REACTOME innate immune system, and tumorigenesis hallmarks such as estrogen response late and KRAS signaling down (Figures 5D,E; Supplementary Table S6B,C). Furthermore, the GSVA algorithm suggested that samples in Cluster A were highly enriched in immune and metabolic hallmarks such as hedgehog signaling, pancreas beta cells, and fatty acid metabolism. In Clusters B and C, samples were highly enriched in proliferation functions such as the mitotic cell cycle, hypoxia, and EMT process (Figure 5F, Supplementary Table S6D). Next, the generalized linear model Cox regression model was used to test the contribution of each function to risk of prognosis for patients with ccRCC (Figure 5G). These results indicated that inflammatory response signaling has a positive effect on prognosis, while the mitotic cell cycle and IL6/JAK/STAT3 signaling are prominent risk factors for ccRCC patients. Based on these results, we hypothesized that IL6/JAK/STAT3 signaling or proliferative phenotype could be a factor leading to the poor prognosis of Clusters B and C.

### DNA variation landscape of immunophenotyping clusters

**FIGURE 6:**
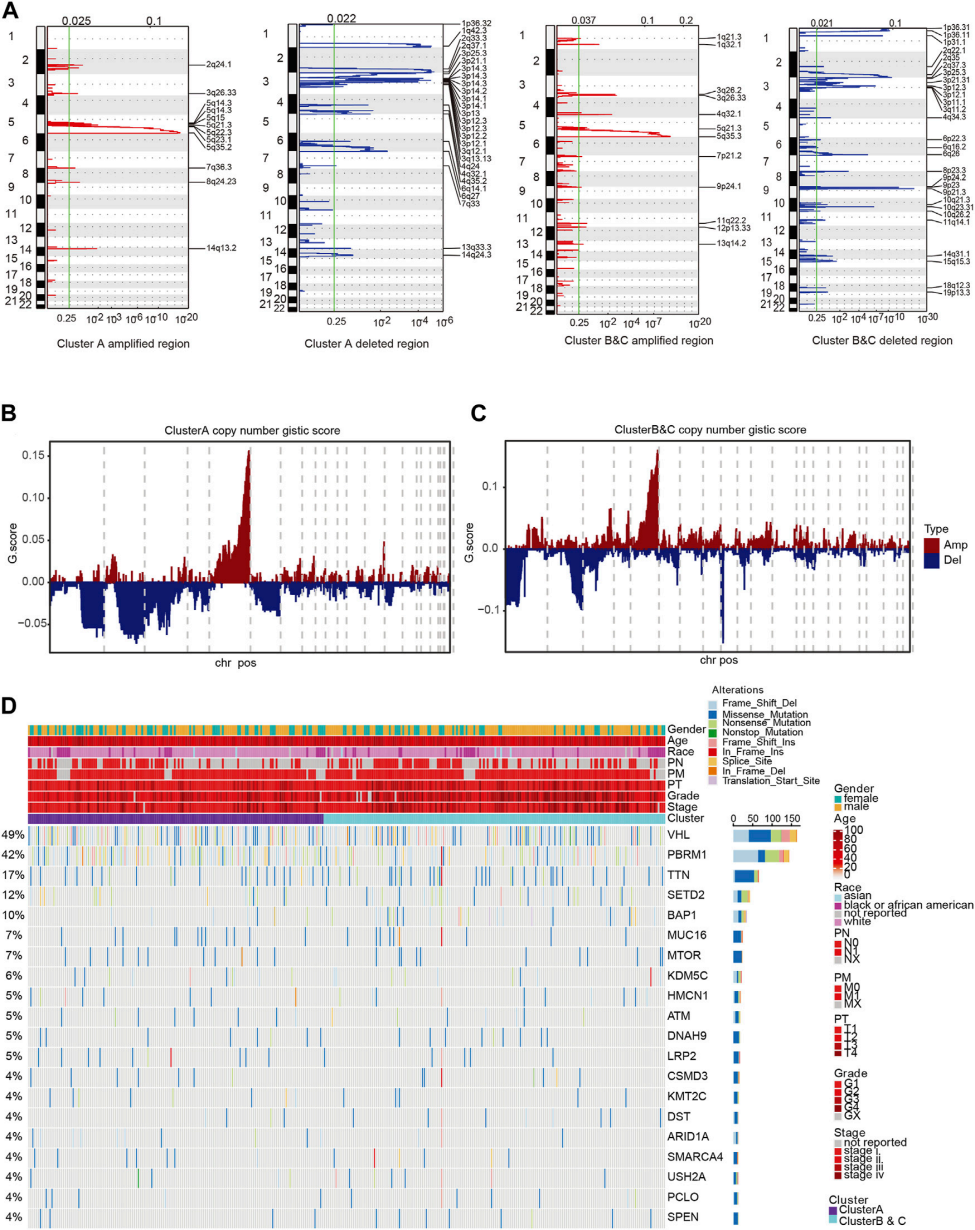
Clinicopathological characteristics of immunophenotyping clusters. **(A)** Differences of various clinical indicators in different immunophenotyping clusters. **(B, C)** Methylation and mRNA expression level of CDKN2A in immunophenotyping clusters. **(D)** Total number of mutations in immunophenotyping clusters. **(E–I)** Phenotypic indicators, such as gender, age, tumor stage, smoking status, MSI, resection, and biopsy site, in the immunophenotyping clusters. **(J)** Patients in immunophenotyping clusters distributed according to AJCC stages.

To further explore DNA variation profiles of the two subgroups, we analyzed the differences in single-nucleotide polymorphisms (SNPs) and CNVs between groups. We found marked differences between copy number amplification and deletion in the two subgroups (Figure 6A). Amplified regions in Cluster A were largely located in 5q11.4, 5q21.3, and 5q35.2, while deleted regions were mainly located in 1q42.3, 2q37.1, 3p14.3, and 6q27. Amplified regions in Clusters B and C were largely located in 3q26.33, 5q21.3, and 5q35.3, which is similar with Cluster A, while deleted regions were mainly located in 1p36.11, 3p25.3, 3q12.3, 9q21.3, and 10q23.31, which were different compared with Cluster A. Additionally, there were significant differences in the Gistic score of the two groups (Figures 6B,C; Supplementary Table S6E,F). We next performed clustering analysis between the immunophenotyping clusters based on SNP, genes with frequent mutations or alterations, and clinical characteristics (Figure 6D). The results indicated that Clusters B and C were accompanied with advanced clinical indicators and frequent TTN, SETD2, and BAP1 gene mutations.

### Immunotherapy efficacy analysis of immunophenotyping clusters

**FIGURE 7:**
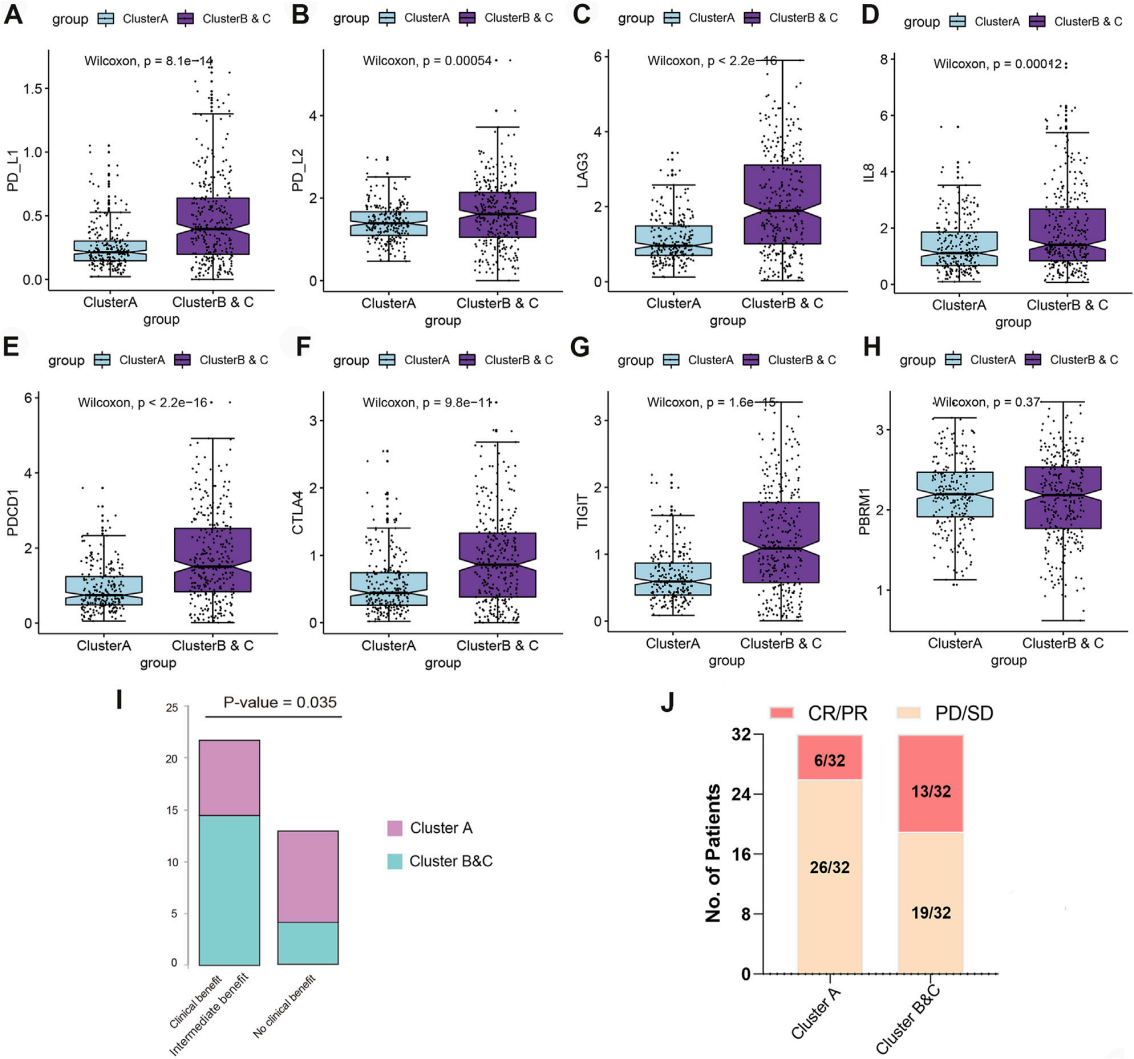
Immunotherapy efficacy analysis of immunophenotyping clusters. **(A–H)** Expressions of immune checkpoint molecules, including PD-L1, PD-L2, LAG3, IL-8, PDCD1, CTLA-4, and TIGIT, and PBRM1, were compared using Students’ *t* test. **(I)** A total of 35 ccRCC patients receiving ICTs from the CA209-009 cohort with RNA-seq data and clinical responses to treatment. **(J)** Response status in 64 ccRCC patients receiving ICTs alone or combined with TKIs from the FUSCC cohort.

To further investigate the predicted responses of immunophenotyping clusters to ICTs, we compared immune checkpoint gene expressions and found that expressions of PD-L1, PD-L2, LAG-3, IL-8, PDCD1, CTLA-4, and TIGIT were significantly elevated in Clusters B and C compared with Cluster A, suggesting an immune-infiltrated TME of ccRCC (Figures 7A–G). No differences in PBRM1 expression were observed in the two subgroups (Figure 7H). We then enrolled 35 ccRCC patients receiving ICTs from the CA209-009 cohort with specific RNA-seq data and clinical response data. Patients in Clusters B and C were significantly inclined to clinical or intermediate benefit (*n* = 22) rather than the no clinical benefit group (*n* = 13) (*p* = 0.035; Figure 7I). Moreover, after grouping 64 ccRCC patients receiving ICTs alone or combined with TKI in the FUSCC cohort, we found prominently increased ccRCC patients from Clusters B and C (13/32) with CR/PR status than patients in Cluster A (6/32) (Figure 7J).

Prognostic implications of immunophenotyping clusters in 360 ccRCC patients from the FUSCC validation cohort.

**FIGURE 8:**
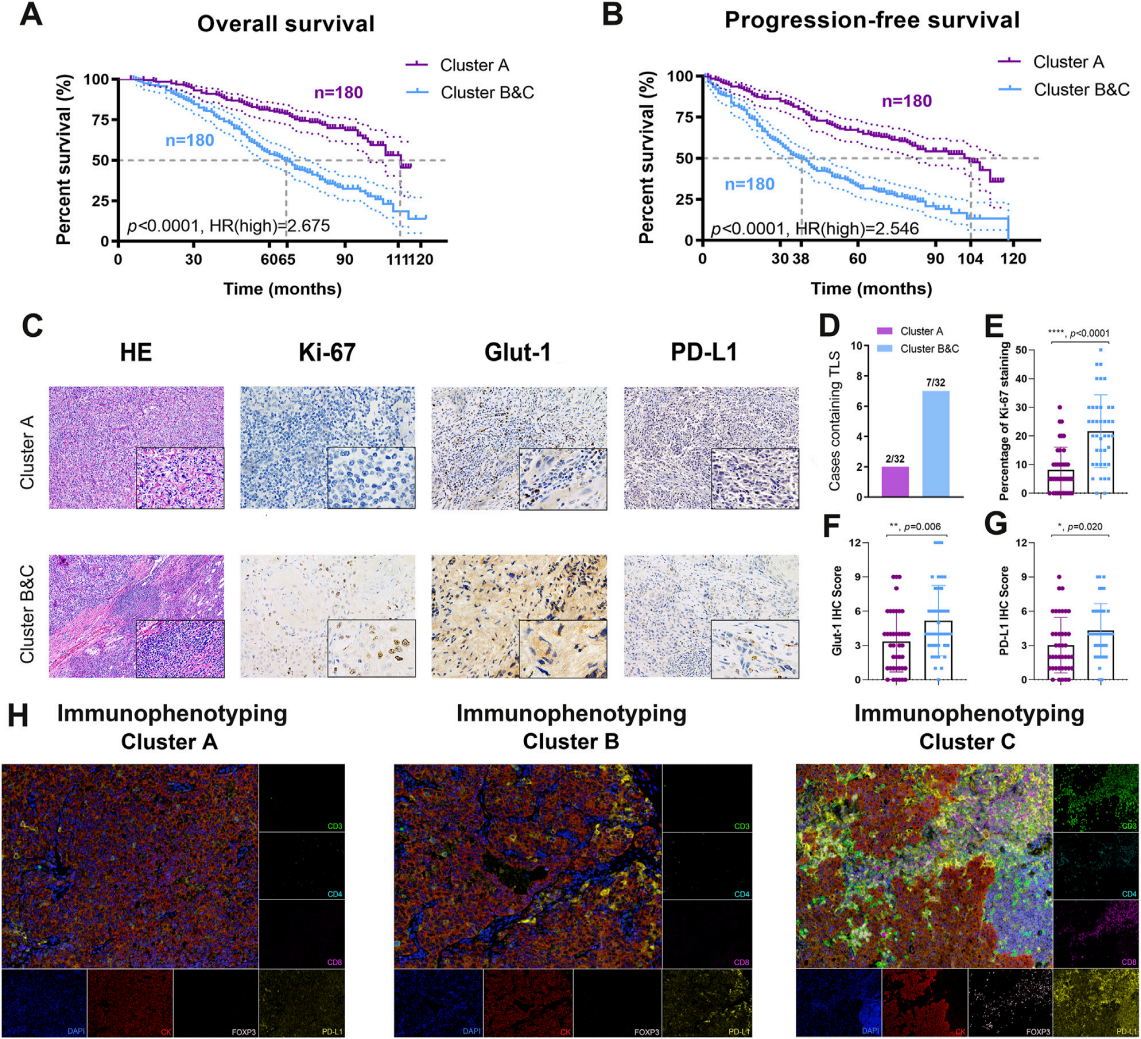
Prognostic implications and TME characterizations of immunophenotyping clusters in a real-world validation cohort. **(A, B)** A total of 360 ccRCC patients with long-term follow-up information from the FUSCC cohort were enrolled to identify the prognostic value of immunophenotyping clusters in the validation cohort. **(C–G)** Characteristics of the tumor microenvironment between immunophenotyping clusters were explored using **(H, E)** and immunohistochemical staining analysis in different clusters. **(H)** Opal multi-label IHC staining was used to identify the abundance of CD^8+^ T cells, CD^4+^FOXP^3+^ Treg cells, and CD^3+^CD^4+^CD^8+^ TCRn immune cell infiltration and PD-L1 expression in clusters.

Although immunophenotyping clusters markedly defined the poor prognosis of ccRCC patients in the training cohort (TCGA, *n* = 531) and testing cohort (ICGA, *n* = 91), large-scale real-world validation evidence was required to confirm the clinical translational value. We thus identified immunophenotyping clusters of 360 ccRCC patients with long-term follow-up information from the FUSCC validation cohort and performed survival analysis (Supplementary Table S7). The results showed that Clusters B and C significantly predicted worse OS compared with Cluster A (*p* < 0.0001, HR = 2.675). The median OS time in Clusters B and C was 66 months compared with 11 months in Cluster A (Figure 8A). In addition, Clusters B and C significantly correlated with poor PFS compared with Cluster A (*p* < 0.0001, HR = 2.822). The median PFS in Clusters B and C was 38 months, while that in Cluster A was 104 months (Figure 8B).

### The TME characterizations differ in immunophenotyping clusters

To provide more experimental evidence for clinical translation, we explored characteristics of the TME in the immunophenotyping clusters. After identification and classification of 64 ccRCC samples receiving ICTs in the FUSCC cohort, we performed H&E and immunohistochemical (IHC) staining analysis in the different clusters (Figure 8C). We found markedly more cases containing TLS in Clusters B and C (37.5%) than in Cluster A (37.5%), suggesting a relatively immune-enriched microenvironment with increased accumulation of mature tumor-infiltrated lymphocytes, such as CD8^+^ T cells (Figure 8D). IHC analysis revealed a significantly more aggressive malignant phenotype, elevated activated glycolysis effect, and PD-L1 expression in Clusters B and C (Figures 8E–G). After opal multi-label IHC staining, we found an increased abundance of CD4^+^FoxP3^+^ Treg cells, CD8^+^ T cells, and CD-predicted favorable response to ICTs for patients with 3^+^CD4^+^CD8^+^ TCRn immune cell infiltration in immune-excluded Clusters B and C compared with immune-desert Cluster A (Figure 8H). Besides, the expression level of PD-L1 was also significantly increased in the Cluster B and C group. Overall, the findings suggested that the pro-tumorigenic Clusters B and C may be associated with an immune-enriched TME and the ccRCC.

## Discussion

This study identified three immunophenotyping clusters in ccRCC with gradual levels of immune infiltration using 758 immune-related genes. As an immune-hot Cluster, Clusters B and C were associated with worse prognosis independent of known clinicopathological indicators, such as myeloid infiltration score, immune score, dryness index score, and mutation. The relatively immune-hot Clusters B and C showed a transcriptional signature indicative of pro-tumorigenic immune infiltration in tumors, and these patients showed significantly worse survival compared with the immune-cold Cluster A. To improve the clinical translational value of the model, we constructed a logistic regression algorithm and identified 26 immune genes constituting a prognostic predictor for subgroup classification. In addition to the distinct immune cell infiltrations in immunophenotyping, there were significant differences in SNVs and CNVs, suggesting a high degree of genetic heterogeneity between the subgroups. We identified two mutually exclusive aggressive tumor phenotypes in ccRCC. Through phenotypic analysis, we found that proliferation and mitotic cell cycle and IL6/JAK/STAT3 signaling were risk factors for Clusters B and C, and multiple metabolic pathways contributed to the survival benefits of Cluster A. Furthermore, the expressions of multiple immune checkpoint molecules, such as PD-1, PD-L1, PD-L2, CTLA-4, and TIGIT, were significantly increased. Importantly, Clusters B and C predicted favorable outcome in 64 ccRCC patients receiving ICTs in the FUSCC cohort. In 360 ccRCC patients in the FUSCC cohort, Clusters B and C significantly predicted worse prognosis compared with the immune-cold Cluster A. After immunophenotyping of ccRCC was confirmed, significantly abundant tertiary lymphatic structures, aggressive phenotype, elevated glycolysis and PD-L1 expression, higher abundance of CD8^+^ T cells and CD4^+^ FOXP3^+^ Treg cells, and M1 macrophage cell infiltration was found in the immune-infiltrated Clusters B and C compared with immune-excluded Cluster A. Therefore, under a paradigm of targeted therapies, such as TKIs, two of the clusters that are “immune-hot” exerted poorer prognosis but might be uniquely responsive to immune checkpoint blockade, thereby improving treatment outcomes for ccRCC patients.

Previous studies have demonstrated that molecular classification of ccRCC showed a prognostic impact in patients treated with VEGFR-TKIs (Beuselinck et al., 2018). The molecular ccrcc1–4 classification of metastatic ccRCC revealed a high predictive value with a significantly higher PFS and OS in patients who received targeted therapy with sunitinib (Verbiest et al., 2018). Mutation of PBRM1 and tumor mutation burden were significantly correlated with poor and good outcome, respectively, which is clinically instructive for the application of molecular targeted therapy and ICTs (Kapur et al., 2013). A previous investigation identified 34 prognosis-related genes through analyzing RNA sequencing expression data and constructed a classifier that divides ccRCC patients into low- (ccA) and high-risk (ccB) groups (Brannon et al., 2010). When the ccrcc1–4 classifier was used to verify the ccA and ccB clusters, a high degree of similarity was found between ccrcc2 and ccA clusters, as well as ccrcc1/4 and ccB clusters (de Velasco et al., 2017). Different from other tumors, in ccRCC, non-synonymous mutations, neoantigens, insertions, or deletions caused by chromosomal structural changes and somatic CNVs were not associated with the efficacy of PD-1 inhibitors (Braun et al., 2020). In addition, the higher level of CD8^+^ T cell infiltration in ccRCC was associated with a poorer prognosis, which was also observed in patients from the Cluster B and C group in this study (Darrow et al., 2020; Black and McGranahan, 2021). However, a large amount of evidence has suggested that not all TMB-high solid tumors are sensitive to ICTs, and high tumor neoantigens are not necessarily accompanied by an increased abundance of CD8^+^ T cell infiltration (McGrail, 2021). Therefore, further tumor type-specific studies are warranted in investigating biomarkers for ICTs.

This study is the first that accurately groups the immune microenvironment in the ccRCC microenvironment. We found that the immune-hot Clusters B and C have pro-tumorigenic immune infiltration and a significantly worse survival than the immune-cold Cluster A, which can be implemented as a novel independent prognostic indicator, highlighting the close relationship between tumor phenotype and the immune contexture. We also developed the construction of classifiers for different immunophenotyping clusters and identified a simple prediction classifier through machine-learning algorithms. The prediction efficiency of the original model was highly consistent, which greatly improved the clinical transformation efficiency. Further phenotypic analysis and functional annotation revealed two mutually exclusive invasive tumor phenotypes in ccRCC: one is related to mitotic cell cycle process, and the other is related to metabolism, suggesting heterogeneity between the ccRCC immunophenotyping clusters. In addition, a high-quality signature for ccRCC to predict the efficacy of immunotherapy was developed. A large amount of evidence in this study shows that the new immunophenotyping of ccRCC significantly predicts the response to ICTs. Besides, describing the correlation between TLS and the clinical benefit of cancer patients, indicating that TLS may be a prognostic and predictive factor, could arouse strong interest in studying the role of TLS in ccRCC.

This study had several limitations. First, this study has not clarified the underlying mechanism of the immunophenotyping clusters of ccRCC. Our future studies will include ccRCC specimens receiving ICTs to explore differences in the immune environment and intratumoral heterogeneity of TME between clusters. Second, although the classifier was constructed and validated using multiple public and real-world datasets, because of the limitation of retrospective analysis, further multicenter studies and prospective trials are warranted for clinical application for patients with ccRCC.

## Conclusion

This study described immunophenotyping clusters that improve the prognostic accuracy of the immune contexture in the ccRCC microenvironment. The immune-hot Clusters B and C showed a transcriptional signature indicative of pro-tumorigenic immune infiltration and significantly worse outcome than the immune-cold Cluster A. Our discovery of novel independent prognostic indicators in ccRCC highlights the relationship between tumor phenotype and the immune contexture.

## Data Availability

The datasets presented in this study can be found in online repositories. The names of the repository/repositories and accession number(s) can be found in the article/Supplementary Material.

## Abbreviations

ccRCC, clear cell renal cell carcinoma; CI, confidence interval; CNV, copy numbers variation; DEGs, differentially expressed genes; EMT, epithelial mesenchymal transitions; FUSCC, Fudan University Shanghai Cancer Center; GSEA, gene set enrichment analysis; HE, hematoxylin-eosin; HR, hazard ratio; ICTs, immune checkpoint therapies; IHC, immunohistochemistry; MSI, microsatellite instability; RCC, renal cell carcinoma; SNP, single nucleotide polypeptides; TLS, Tertiary lymphoid structure; TME, tumor microenvironment; TKIs, tyrosine kinase inhibitors.
