# Supplementary figures and images for: Prognostic Immunophenotyping Clusters of Clear Cell Renal Cell Carcinoma Defined by the Unique Tumor Immune Microenvironment
Source: Front Cell Dev Biol. 2021 Dec 6;9:785410. doi: 10.3389/fcell.2021.785410 (PMC8685518; doi:10.3389/fcell.2021.785410)

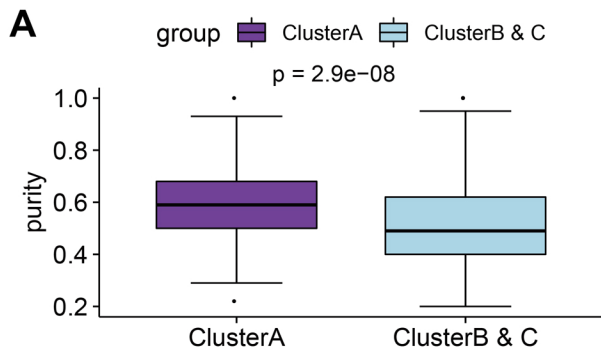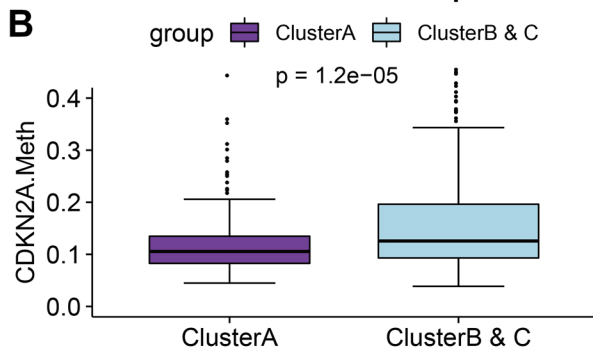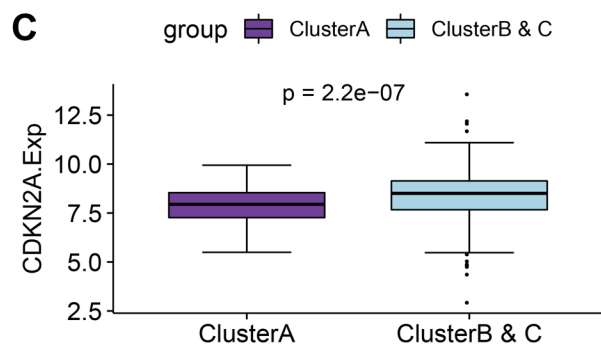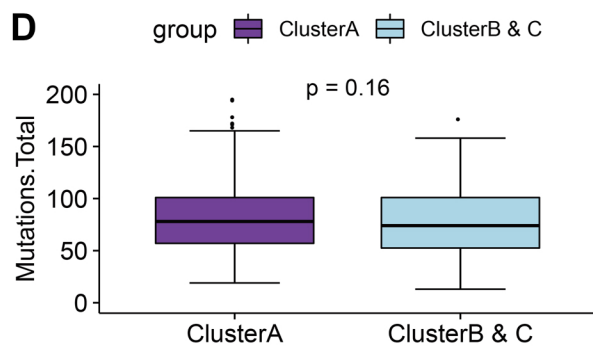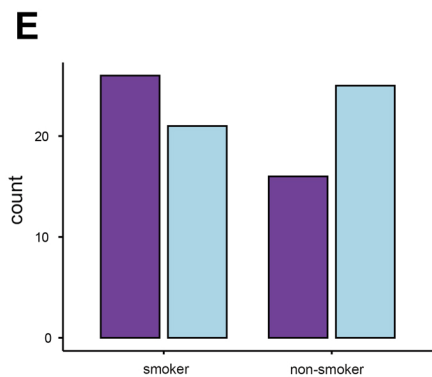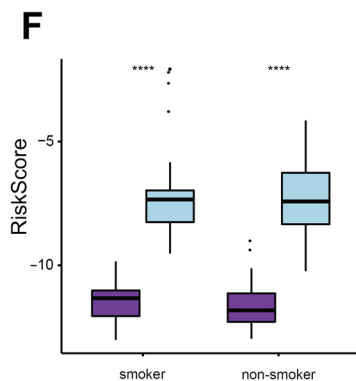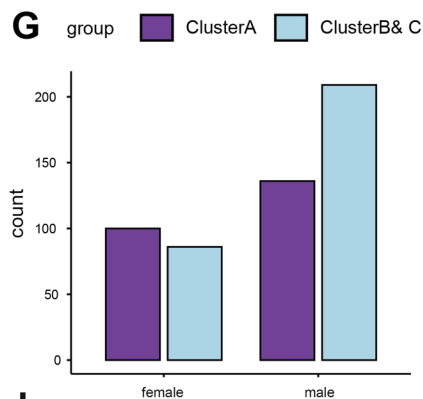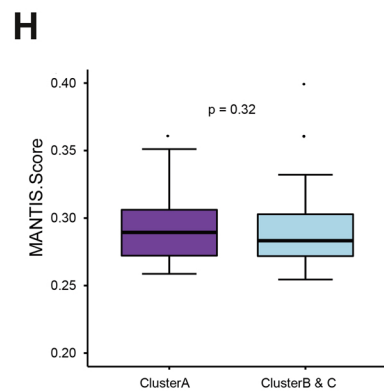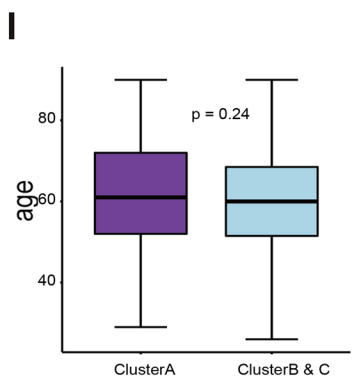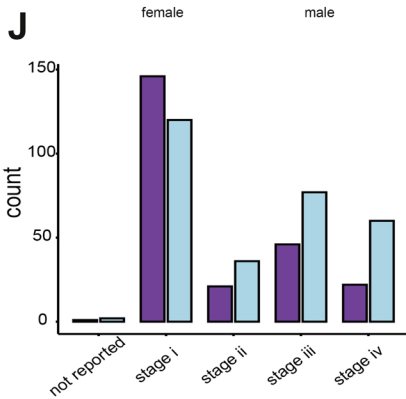

Supplement: Supplementary file 3 [file Image2.pdf]

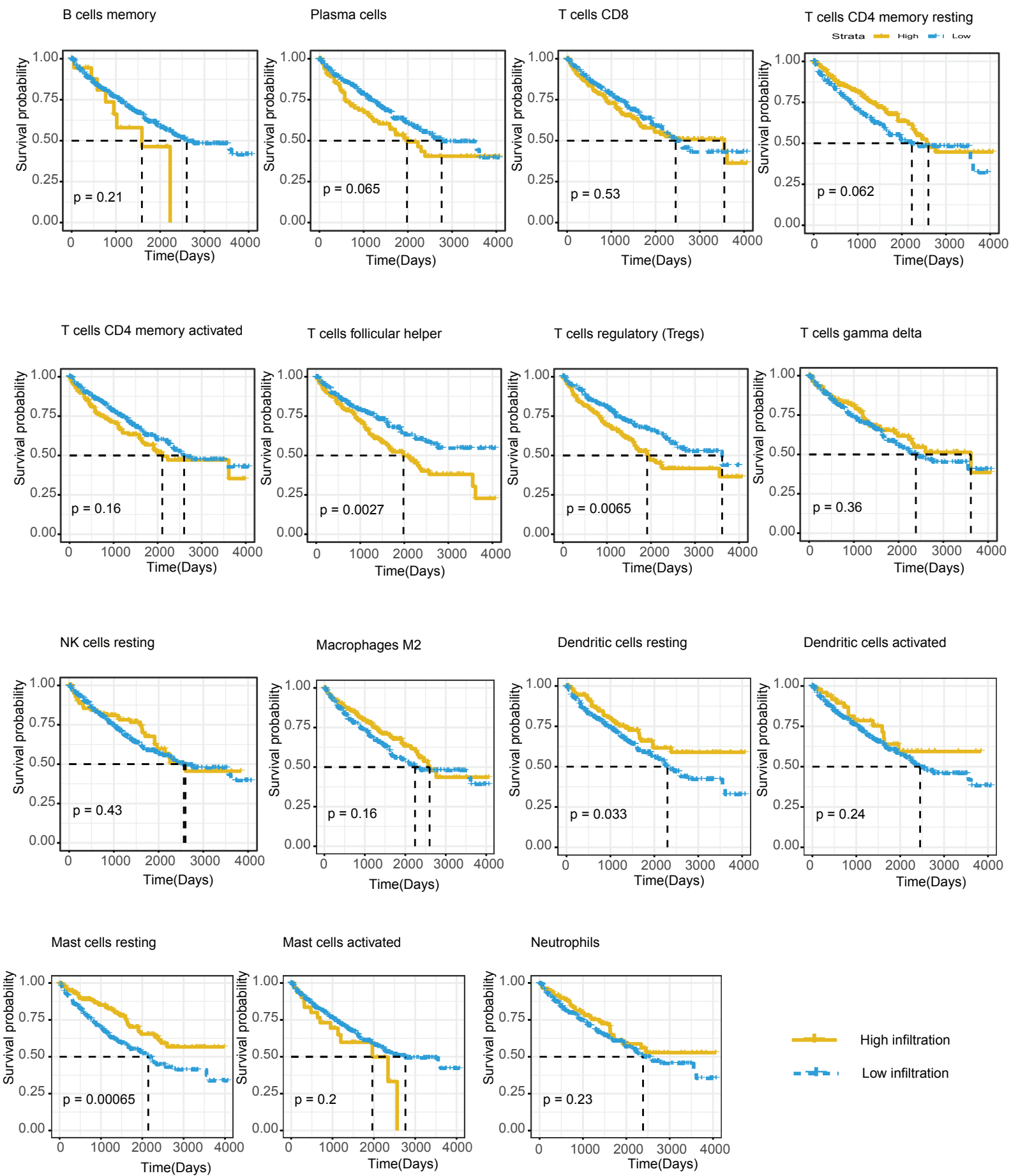

Supplement: Supplementary file 5 [file Image3.pdf]

OS

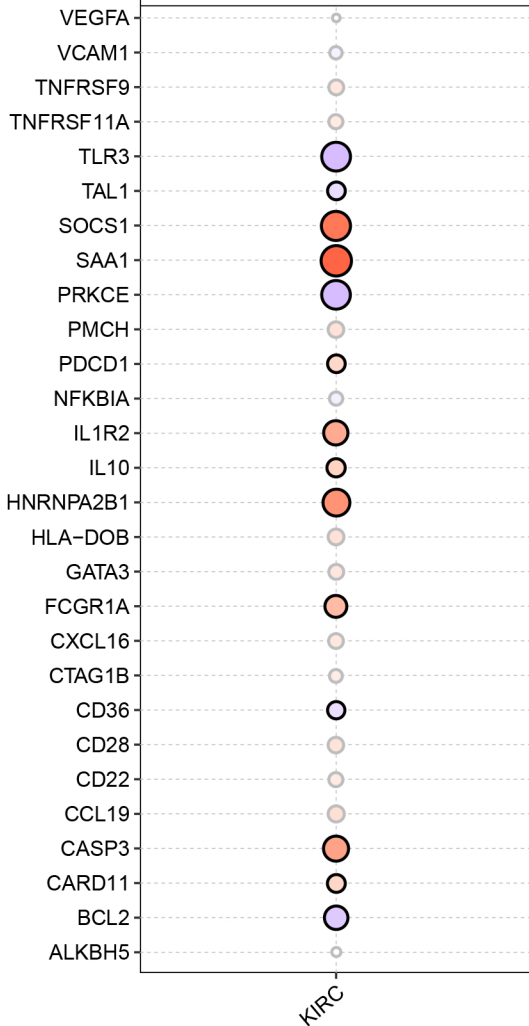

KIRC

Supplement: Supplementary file 10 [file Image1.pdf]
